# Measuring and modelling occupancy time in NHS continuing healthcare

**DOI:** 10.1186/1472-6963-11-155

**Published:** 2011-06-29

**Authors:** Salma Chahed, Eren Demir, Thierry J Chaussalet, Peter H Millard, Samuel Toffa

**Affiliations:** 1 Department of Information Systems and Computing, University of Westminster, 115 New Cavendish Street, London, W1W 6UW, UK; 2 Department of Marketing & Enterprise, University of Hertfordshire, de Havilland Campus, AL10 9EU, Hertfordshire, UK; 3 Geriatric, St George's University of London, Cranmer Terrace, London, SW17 0RE, UK; 4 Software Development Unit, HPA, Centre for Infections, 61 Colindale Avenue, London, NW9 5HT, UK

## Abstract

**Background:**

Due to increasing demand and financial constraints, NHS continuing healthcare systems seek to find better ways of forecasting demand and budgeting for care. This paper investigates two areas of concern, namely, how long existing patients stay in service and the number of patients that are likely to be still in care after a period of time.

**Methods:**

An anonymised dataset containing information for all funded admissions to placement and home care in the NHS continuing healthcare system was provided by 26 (out of 31) London primary care trusts. The data related to 11289 patients staying in placement and home care between 1 April 2005 and 31 May 2008 were first analysed. Using a methodology based on length of stay (LoS) modelling, we captured the distribution of LoS of patients to estimate the probability of a patient staying in care over a period of time. Using the estimated probabilities we forecasted the number of patients that are likely to be still in care after a period of time (e.g. monthly).

**Results:**

We noticed that within the NHS continuing healthcare system there are three main categories of patients. Some patients are discharged after a short stay (few days), some others staying for few months and the third category of patients staying for a long period of time (years). Some variations in proportions of discharge and transition between types of care as well as between care groups (e.g. palliative, functional mental health) were observed. A close agreement of the observed and the expected numbers of patients suggests a good prediction model.

**Conclusions:**

The model was tested for care groups within the NHS continuing healthcare system in London to support Primary Care Trusts in budget planning and improve their responsiveness to meet the increasing demand under limited availability of resources. Its applicability can be extended to other types of care, such as hospital care and re-ablement. Further work will be geared towards updating the dataset and refining the results.

## Background

In the UK, Continuing Care (CC), also known as Long-Term Care (LTC), is provided to people aged 18 or over with physical or mental health needs over an extended period of time or in the period immediately prior to death [1]. CC conditions may arise in a situation of disability, accident or illness, which cannot be cured, but only treated by medication and other therapies. Many individuals receiving CC have long-term conditions and are intensive users of health and social care services; and their numbers are expected to rise [2].

Continuing care places are generally provided by the National Health Service (NHS) (i.e. publicly-funded healthcare system) and/or Local Authorities (LAs) (i.e. administrative office running a defined area), and are available through various settings (e.g. hospital, care home, hospice, home care). Patients with continuing care conditions may require healthcare services provided through the NHS (e.g. Primary Care Trusts, NHS Trusts, Mental Health Trusts) and/or social and community care services provided by LAs (e.g. borough, county councils). The National Framework published in 2007 [1], guidance helping to improve both the consistency and understanding of one single national approach towards NHS continuing healthcare in England, clarifies the eligibility criteria for NHS Continuing Healthcare and supports the decisions on how best to meet patients' needs. In fact, the needs for NHS care are assessed to decide about the eligibility of the patient. Patients whose main needs relate to their health are those eligible for funded NHS care. Patients who are not eligible for funded NHS care may qualify for a joint package of continuing care, i.e. shared responsibility between the NHS and the LA for providing the care.

Given the limited availability of resources, a focus on CC issues will become necessary for managing the ageing society. It is interesting for the NHS and LA bodies to understand the behaviour of the CC system and to estimate future admissions. Unfortunately, it is challenging to estimate the annual budget due to uncertainties about the movements of current residents in the CC system and future demand. Information on demand will provide the NHS and local authorities with an effective management strategy and improvement in their responsiveness to meet the increase in demand. The aim of this paper is to analyse the occupancy time relative to patients already in the NHS continuing care system, in particular, to determine how many patients will stay in the system (demand projection) and for how long (survival model). To achieve this, we adapt a methodology based on LoS modelling, which was previously tested and validated within the context of a local authority in England.

This paper is organised as follows. The methods section introduces the LoS modelling approach and describes the NHS continuing healthcare dataset. The results section describes the survival patterns within the NHS continuing healthcare system, which are further used to estimate the number of patients that are likely to be still in care after a period of time. These findings are analysed in the discussion section. Finally, a summary of the results and some perspectives are provided.

## Methods

### Survival model and demand projection

In a letter to the British Medical Journal [3] in 1963, Struthers reported that equations with two exponentials fitted well discharge length of stay in a department of geriatric medicine providing short and long stay patients. He also argued that careful attention to the genuine social and medical needs of potential long stay patients increases admissions by decreasing the number of long stay patients. This observation has motivated authors to develop a theory of flow [4] and a mathematical model [5], which both explained why exponential equations fitted well the pattern of length of stay in departments of geriatric medicine.

**Figure 1:**
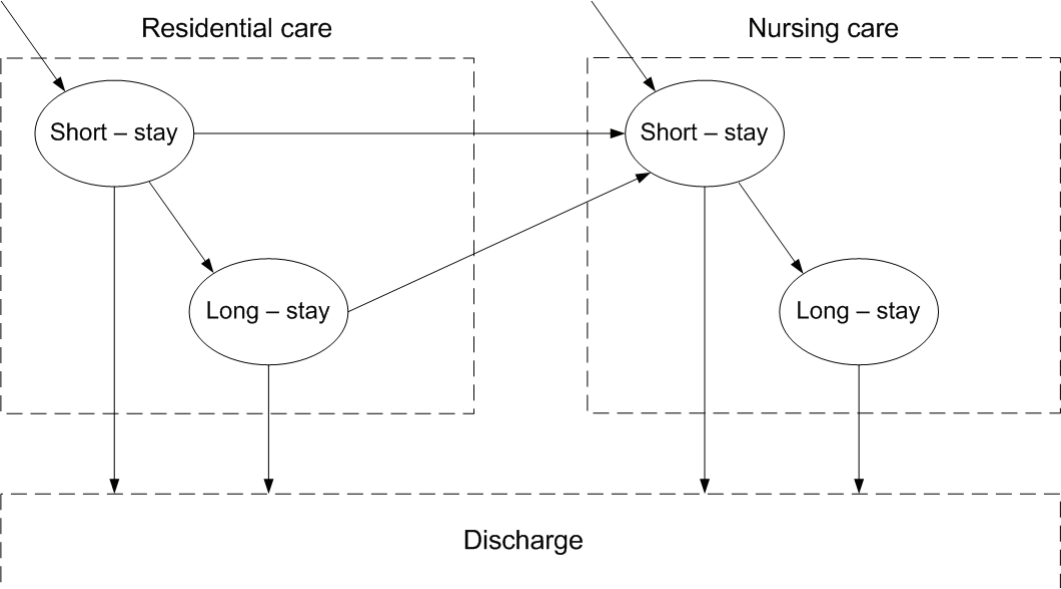
**Model for the movements of residents in institutional long-term care adapted from **[7]. Upon entering either type of care residents follow a short stay state [or period] after which they are either discharged or enter a long-stay state, which may last for months, if not years. In addition residents in residential care may also transfer to nursing care upon leaving their short or long stay state. All residents are eventually discharged.

Xie et al [6] examined the survival pattern of elderly residents in institutional long-term care (ILTC) and the impact of residents' attributes on length of stay. The developed model captures the survival and movement patterns of LTC residents placed in institutional care (e.g. residential care, nursing care) and funded by the local authority. The model also extends a model developed previously by the authors [7] by handling left-truncated in addition to right-censored data. A continuous time Markov model of the flow of elderly residents within and between residential and nursing care assumes that residents may go through two conceptual states (i.e. not observable) - short-stay and long-stay states - before discharge, predominately by death [7] (see, Figure 1). For example, at any given time we could observe that a person is in residential home care but we do not know whether s/he is in a short or long stay sate. This intuitive assumption was also validated with empirical evidence using the Akaike information criterion (AIC) and the Bayesian information criterion (BIC) to select the model which gives best fit with least complexity [number of states]. Residents admitted to residential care might stay for a short period of time (short-stay state) and could either be discharged or moved to nursing care. Otherwise, they would stay for a longer period of time (long-stay state) and then be discharged or moved to nursing care. Similarly, Faddy et al used in [8] a Markov model to represent the process of hospital stay. They demonstrated that the resulting distribution provides a good fit to the patients' length of stay data unlike gamma and log normal distributions.

Based on the survival model presented in [7], Pelletier et al developed a costing framework that translates survival inputs and gross unit costs into total cost, which is the sum of costs of maintaining each resident during the forecasting period [9]. In addition, they examined the impact of potential changes in pricing policies (i.e. increase in unit prices of care in residential care and nursing care) on local authority's budget planning. As a result of the aforementioned works, the Health and Social Care Modelling Group (HSCMG) in collaboration with the Care Services Efficiency Delivery (CSED, Department of Health) developed a software implementation of the forecasting framework [10], known as FLoSC (Forecasting Length-of-Stay and Cost) (cf. http://www.healthcareinformatics.org.uk/flosc/). The presented version of the software tool was a prototype which was tested in collaboration with an English borough. It guides users through a set of screens of options in a familiar wizard fashion. Over 100 Local Authorities have downloaded FLoSC to date and are making use of the tool.

We adapt this approach and apply a length-of-stay model similar to [6], which handles both left truncated and right censored data, to the case of the NHS continuing healthcare system. The main differences in the model are the need for more than two conceptual states to achieve suitable model fit and the fact that movements between types of care, which rarely occur in continuing care, do not need to be considered.

### NHS Continuing healthcare data

At the national level, the recent NHS World Class Commissioning (WCC) competency framework outlines how Primary Care Trusts (PCTs) (i.e. corporations that provide or commission primary and community care services, and that are involved in the commissioning of secondary care services) should be commissioning their services [11]. To respond to the WCC initiative, PCTs will definitely need help in forecasting the continuing care demand. Although initially developed for local authorities, the previously described approach (cf. subsection "Survival model and demand projection") may also be applicable for primary care trusts since patients with continuing care conditions may require healthcare services via NHS organisations and/or social and community care services through local authorities. In the following section, we will apply this approach to the NHS continuing healthcare field using London PCTs data to forecast existing patients' length-of-stay and estimate the number of patients who are likely to survive.

PCTs are responsible for providing continuing healthcare services for a variety of care groups, i.e. Physically Frail (PF), Palliative, Physically Disabled Adult (PDA), Organic Mental Health (OMH), Functional Mental Health (FMH) and Learning Disability (LD). These healthcare services are provided in placement, i.e. institutional care [residential and nursing], or home care, i.e. care and support at patient's home. Residential care is for people who cannot continue living in their own home, even with support from home care services. It can include personal care, such as eating, washing and dressing. Nursing care is provided by registered nurses in a care home.

An anonymised dataset containing information for all funded admissions to placement and home care in the NHS continuing healthcare system of 26 London PCTs (out of 31 London PCTs) was collated and provided by the NHS London Procurement Programme (LPP). The dataset consists of secondary administrative data routinely collected by PCTs for financial purpose. A confidentiality agreement was signed between the University of Westminster and the LPP. Initially, the dataset contained 13700 records. Our interest lies in the occupancy time in placement and home care separately, and not movements between the two, which rarely occur anyway. As a result, if a patient has a footstep in both placement and home care, these are assumed to be independent, and a different ID is assigned to each record. The period from 1 April 2005 and 31 May 2008 was used to reconcile datasets provided by different PCTs, which brought the number of records to 13265, i.e. cases where patients were admitted after the end, or discharged before the start, of that period were removed from the dataset. The dataset contained the following information: care group, type of care, date of admission, date of discharge, discharge reason, age at admission and weekly cost rate. Since PCTs do not necessarily have the same information system, data coding was checked and unified. After removing records with missing type of care, length of stay and discharge reason, the dataset reduced to 11289 records (we obtain 12537 records by deleting only records with missing type of care, cf. Table 1). Table 1 indicates in italics the numbers of records after deleting missing type of care, length of stay and discharge reason data. Between parentheses are the numbers of records after removing missing type of care.

**Table 1:** Numbers of records related to patients staying in the NHS continuing care system

	*Type of care*		*Total*
	
*Care groups*	Placement	Home Care	
Physically Frail	*2123 *(2267)	*604 *(648)	*2727 *(2915)

Palliative	*2315 *(2478)	*2973 *(3349)	*5288 *(5827)

Physically Disabled Adult	*621 *(659)	*333 *(357)	*954 *(1016)

Organic Mental Health	*1159 *(1245)	*147 *(159)	*1306 *(1404)

Functional Mental Health	*429 *(509)	*12 *(41)	*441 *(550)

Learning Disability	*323 *(529)	*33 *(49)	*356 *(578)

*Total*	*6970 *(7687)	*4102 *(4603)	*11072 *(12290)

*Total including missing care group data*	*7174 *(7919)	*4115 *(4618)	*11289 *(12537)

On 1 April 2005, there were 1224 patients (986 in placement and 238 in home care) in NHS continuing healthcare. During the three-year period there were 10065 admissions (6189 to placement and 3876 to home care). At the end of the period, there were 3761 patients still in care (2803 in placement and 958 in home care).

**Figure 2:**
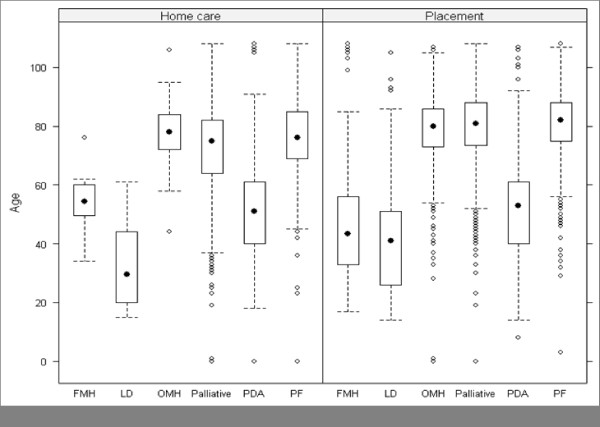
**Box plots of age per type of care and per care group**. Note that as a number of patients have a footstep in both placement and home care, they appear in box plots relative to both types of care.

Figure 2 illustrates box plots of patient age in the NHS continuing healthcare system. These box plots show patients' lowest estimated (lower adjacent limit), first quartile (bottom of the box), median (black circle), third quartile (top of the box) and highest estimated age (upper adjacent limit) for every care group and type of care. Outliers are represented by small white circles. We observe that the age of the majority of FMH, LD and PDA patients are below 60 in both type of care, whereas the age of the majority of OMH, Palliative and PF patients are above 60.

**Figure 3:**
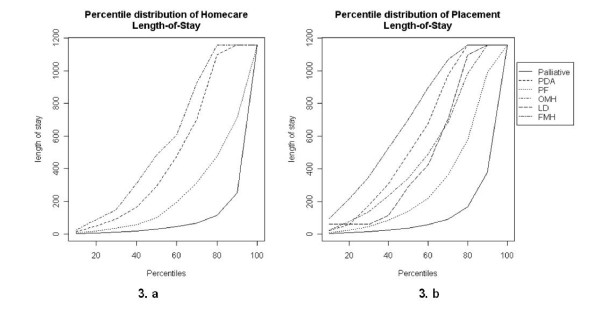
**Percentile distribution of Length-of-Stay per type of care and care group**. PDA: Physically Disabled Adult; PF: Physically Frail; OMH: Organic Mental Health; LD: Learning Disability; FMH: Functional Mental Health.

Figures 3.a and 3.b represent the percentile distribution of length-of-stay for Home care and Placement, respectively. For instance, in Placement, half of Palliative patients are treated within 38 days, whereas half of PDA patients are treated within 493 days. We observe that FMH patients are long stayers compared with the other care groups. Although we have shown the age distribution for FMH and LD, we have decided not to present the length-of-stay distributions for these care groups due to limited number of records (cf. Table 1).

## Results

### Survival patterns

Current models of institutional care have two conceptual states, namely short-stay and long-stay states. However, in practice this may be restrictive as patients from other care systems may experience more than two conceptual states. Having removed this restriction, we observe three states within the NHS continuing healthcare system. These states can be labelled as short-stay, medium-stay and long-stay states.

**Figure 4:**
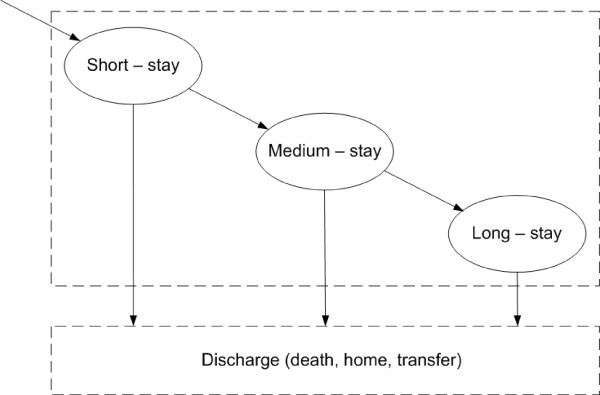
**Conceptual model of residents' movement within a type of care in NHS Continuing healthcare**. Within the NHS continuing healthcare system there are three main categories of patients. Some patients are discharged after a short stay (few days), some others staying for few months (medium stay) and the third category of patients staying for a long period of time (long stay, e.g. years).

Movements between types of care within the NHS continuing healthcare are rare. For this reason, we studied the distribution of length-of-stay only within the types of care. We fitted the model using FLoSC to the observed three year length-of-stay data, and estimated the average length-of-stay per state for placement and home care. We also estimated the proportion of patients discharged from each state and computed the proportion of patients moving between states. Figure 4 illustrates the conceptual movement and survival patterns of residents per type of care at the London-wide level. Note that the discharge (death, home, transfer) destination is an absorbing state, as information concerning outcome concerns the date financial assistance ceased and not the destination.

**Figure 5:**
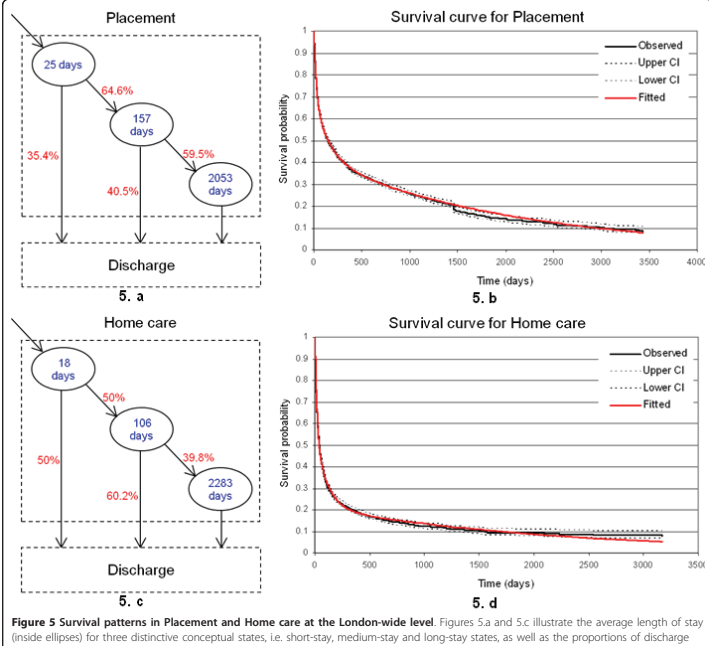
**Survival patterns in Placement and Home care at the London-wide level**. Figures 5.a and 5.c illustrate the average length of stay (inside ellipses) for three distinctive conceptual states, i.e. short-stay, medium-stay and long-stay states, as well as the proportions of discharge and transition (on the arrows) between states for both types of care. Figures 5.b and 5.d illustrate the survival curves for placement and home care, respectively.

In Figures 5.a and 5.c, three distinctive states can be observed for both types of care, i.e. placement and home care. For placement, the average length-of-stay (inside ellipses) for short-stay, medium-stay and long-stay states is estimated to be 25, 157 and 2053 days (more than 5.5 years), respectively. The proportions of discharge and transition between states are on the arrows. Just over a third (35.4%) of the short-stay residents will either die or leave the system alive and the remaining 64.6% will become medium-stay residents in placement. Again 40.5% of the latter category of residents will be discharged and the remaining (59.5%) will stay for a much longer period of time (long-stay state) before discharge. For home care, the average length-of-stay for short-stay, medium-stay and long-stay states is estimated to be 18, 106 and 2283 days (about 6.2 years), respectively. The probability of discharge from home care is higher than placement, with 50% of the residents after an average length-of-stay of 18 days (i.e. discharged from the short-stay state) and 60.2% of the residents from the medium-stay state. At the London-wide level, the average of length-of-stay in placement and home care is 3.7 years and 2.7 years, respectively.

Figures 5.b and 5.d illustrate the survival curves for placement and home care, respectively. A survival curve is a graphical representation of the probability of a resident staying longer than a period of time (x-axis), i.e. the probability that a resident will survive the system past a certain time. There is a close agreement between the fitted survival curve (red solid line) and the observed survival curve (black solid line), hence suggesting that three states capture the overall movement of the NHS continuing healthcare patients. The 95% confidence interval (black dotted lines) suggests a good fit of the data and the reliability of the model.

According to the survival curves (Figures 5.b and 5.d), 50% of the residents in placement will stay for longer than 148 days (about 5 months), 20% of those residents will stay for more than 4.2 years, and 10% of them will still be in care for more than 8 years. We also notice a sudden drop in the survival probability for placement which could be due to an increase in capacity, i.e. new places opened. About 20% of the home care residents will stay for more than 318 days (about 10.6 months), and 10% will stay longer than 4.6 years.

**Figure 6:**
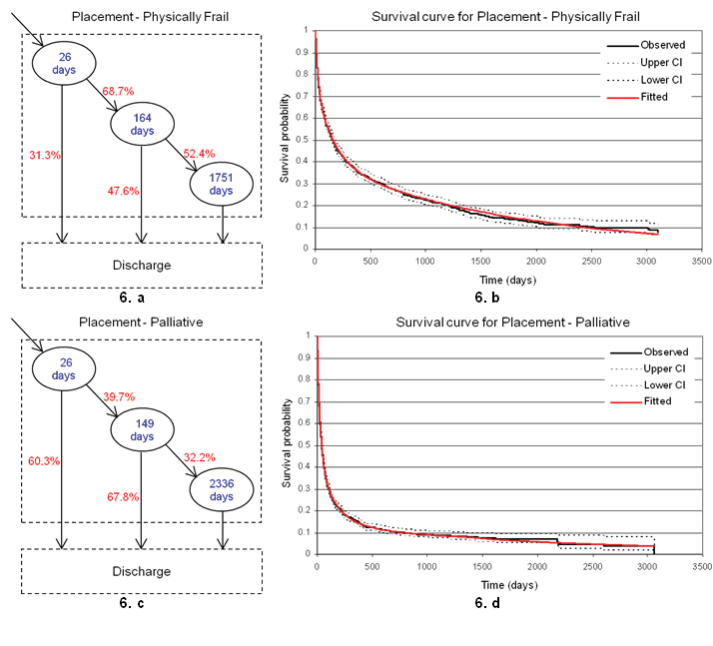
**Survival patterns of Physically Frail and Palliative residents in Placement**. Figures 6.a and 6.c illustrate the three distinctive conceptual states for both types of care. Figures 6.b and 6.d illustrate the survival curves for placement and home care, respectively.

So far we have carried out the analysis for placement and home care patients. However, there are subcategories of types of care, i.e. care groups. For illustration purposes, we re-examine the movement patterns and survival curves of Physically Frail and Palliative patients in Placement. This enables us to study homogenous length-of-stay based clusters, i.e. the modelling is now based on similar groups of patients. Figure 6 illustrates the results at the London-wide level: Placement - Physically Frail (Figures 6.a and 6.b) and Placement - Palliative (Figures 6.c and 6.d). The survival curves show a good fit of the data, since the fitted models are both within the 95% confidence interval. Figures 6.b and 6.d illustrate the diversity in the survival patterns of these care groups. For instance, about 50% (respectively 10%) of Physically Frail patients who have been admitted to placement will stay for longer than 157 days (respectively 6.7 years), whereas 50% (respectively 10%) of those requiring Palliative care will survive beyond 39 days (respectively 2.2 years).

Furthermore, while comparing the movement patterns (see, Figures 6.a and 6.c), we note that the discharge proportions from short-stay and medium-stay states for Physically Frail patients are lower than those of Palliative patients. For Physically Frail patients, about 31.3% of the patients will be discharged from the short-stay state after an average length-of-stay of 26 days and 47.6% will stay for a longer period of time (i.e. discharged from the medium-stay state). However, for Palliative care, around 60.3% will be discharged from the short-stay state after an average length-of-stay of 26 days, and 67.8% will stay for a longer period (medium-stay state). Hence, the majority of Palliative patients left the system within a month. At the London-wide level, for Placement, the average length-of-stay for Physically Frail patients and Palliative patients is 2.9 years and 2.3 years, respectively. Although the discharge proportion for Palliative care is higher than that for Physically Frail care, the difference between the average lengths-of-stay is low (few months). This may be due to fewer Palliative patients (12.8%) staying for a longer period of time (an average of 6.4 years for Long-stay state) before discharge, whereas a higher proportion of Physically Frail patients (36%) is observed to stay for a shorter period of time (an average of 4.8 years for long-stay state).

### Demand projection

In a market based economy, health and social purchasers as well as providers need to know and understand how past decisions influence present and future budget allocations. Moreover, by modelling the impact of past decisions on future opportunities and choices managers in health and social care organisations can understand better the number of patients that are likely to be still in care after a period of time. This information enables decision makers to estimate available capacity, as well as costs of maintaining current patients, etc., hence to allocate resources accordingly, and support planning and management decisions within the NHS continuing healthcare system. Using the survival model, given that there are N patients who are still alive at a particular point in time, we estimate the number of patients who are likely to survive over a specified period of time using the survival model.

For a particular type of care and care group, the probability that a patient will stay in the system beyond ***x ***days given that he/she has already survived ***a ***days is defined by the survival model. For instance, a Physically Frail male patient who has being in care for 392 days (and is currently in Placement at 31 May 2007), has a probability of 0.92 and 0.76 of surviving more than 3 months and one year, respectively. This probability is determined for every patient who is still in care at a particular time, and the sum of these probabilities is used to determine the expected number of patients, who are in care, ***x ***days later [9].

We divided the three year dataset in two parts to test the projection capability of the model. We extracted a two-year dataset (01 April 2005 - 31 May 2007) relating to patients still in care on the 31^st ^of May 2007 from the original dataset (see subsection "NHS Continuing healthcare data"), and we projected the patient numbers for the next year. Patients admitted after, or discharged before, 31^st ^May 2007 were not considered. Hence, on 31 May 2007, there were 2355 patients in placement and 747 patients in home care, cf. Table 2. Note that since there were limited numbers of Learning Disability (LD) and Functional Mental Health (FMH) patients placed in Home care, these records were not included in the dataset.

**Table 2:** Numbers of patients still in care on 31 May 2007

	*Type of care*	
***Care groups***	**Placement**	**Home Care**

Physically Frail	655	177

Palliative	346	330

Physically Disabled Adult	351	145

Organic Mental Health	594	81

Functional Mental Health	265	6

Learning Disability	144	8

*Total*	2355	747

**Figure 7:**
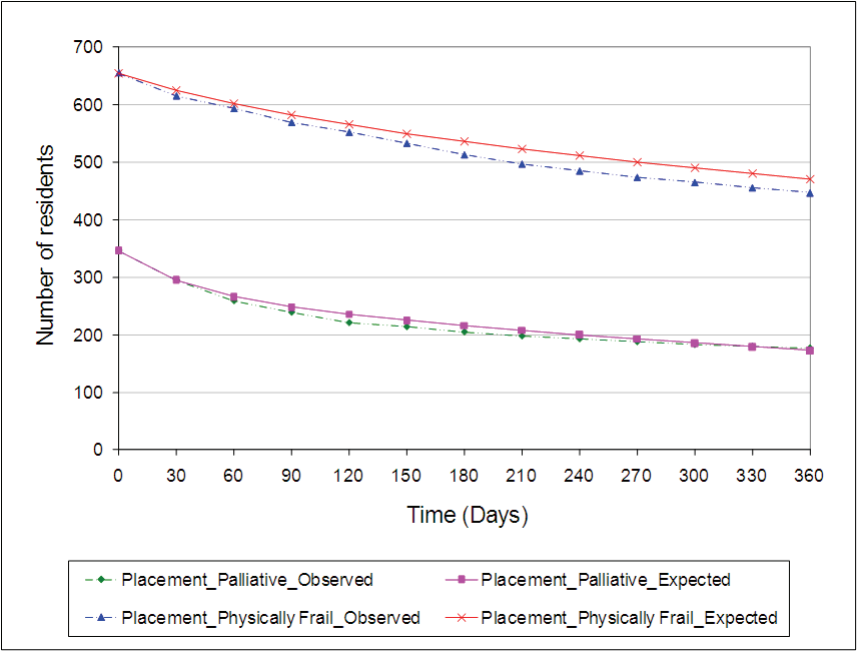
**The observed and the projected monthly number of Physically Frail and Palliative residents in Placement**. Dotted lines represent the observed monthly number of patients; Solid lines represent the expected monthly number of patients.

Figure 7 illustrates the observed and the expected monthly number of Physically Frail and Palliative patients in Placement between 01 June 2007 and 31 May 2008. Dotted lines represent the observed monthly number of patients, whereas the solid lines represent the expected monthly number of patients. The close agreement of the observed and the expected numbers of patients suggests a good prediction model. According to Figure 7, we expect 72% of the Physically Frail cohort and half the Palliative patients to stay in Placement beyond one year. Furthermore, we notice that the number of Palliative patients decrease faster than Physically Frail judging by the survival curves illustrated in Figures 6.b and 6.d.

## Discussion

Local authorities and primary care trusts continue to be faced with the challenges of making best use of resources and evidencing value for money at every opportunity.

Good use of resources is at the heart of efficiency and service outcomes, hence balancing the act between user expectations and the priorities of continuing care services. As a result, planning for the future by matching the resources available with known demands would certainly minimise financial risks and ensuring patient/resident satisfaction. Making critical decisions without an evidence based approach to commissioning services could potentially be highly costly and have a negative effect on the system as a whole. This paper tackled some of the main concerns of the commissioning process within the NHS continuing healthcare system, which is in line with the National Service Framework to ensure integrated health and social care services for older people, e.g. an evidence based approach to capture the existing system (LoS patterns) and forecasting demand such that the results are robust enough to use it as a means to making critical decisions.

In this paper, we analysed the current demand related to residents already in the NHS continuing healthcare system. More precisely, we studied the length-of-stay of patients within a particular type of care (placement or home care) and per care group (PF, Palliative, PDA, OMH, FMH or LD), and predicted their numbers over a given forecast period. Using data provided by 26 London PCTs, the movement of patients were captured within the NHS continuing healthcare system, and we observed that 3 states - short-stay, medium-stay and long-stay states - best described the provider-purchaser system, although only two conceptual length-of-stay states were observed in ILTC system. This difference might be a result of the eligibility criteria specified by the Department of Health's National Framework.

In addition, we noticed some variations in proportions of discharge and transition between types of care as well as care groups. For instance, the proportions of discharge from home care are higher than from placement. The proportions of discharge from short-stay and medium-stay states for Physically Frail patients are lower than those of from Palliative care.

Several unexpected results occurred. For example, clinically Palliative Care is usually considered to be short term, i.e. a stage between life and death, involving pain and symptom control. Yet within the data we analysed palliation exhibited a long stay function too. In fact, we observed that there are two main categories of Palliative patients, where the majority are discharged after a short/medium stay (few months) and the second category of patients staying for a very long period of time (years). More precisely, 10% of patients staying in Palliative care will survive beyond 2.2 years blocking places for a long time. Whether this represents coding or a separate stage after hospice care which is time unlimited is unclear. This observation could lead PCT managers to switch these patients to re-ablement services.

## Conclusions

This study was motivated by the interest of PCTs to use a length-of-stay model in order to support their planning decision making and improve their responsiveness to meet the increase in demand. Although initially developed for local authorities [7], the applied approach is also useful for primary care trusts, since patients with continuing care conditions may require healthcare services through NHS organisations and/or social and community care services through local authorities. In this study, we have examined the movement patterns and survival curves of continuing care patients not only per type of care but per care group as well. This approach enabled us to study homogenous length-of-stay based clusters. Unfortunately, during the data preparation phase, we noticed an unusual length-of-stay distribution for LD and FMH in home care, which was due to the limited number of records, and as a result, these were removed from the analysis. Inclusion of these records would result in inaccurate estimation of the demand, hence mislead decision maker and increase financial risks. Further work will be geared towards updating the dataset, refining the results and possibly including other features.

The proposed model does not cope with more than three states (i.e. short-stay, medium-stay and long-stay states). In some cases, there could be an underestimate of fit, and so the possibility of a fourth state. In this study we have not found such cases, i.e. the model provides good fit to the observed length of stay data, but it could be different for other PCTs, type of cares or care groups.

In addition, this model was only tested for care groups within the NHS continuing healthcare system and needs to be rigorously tested for care groups specific to the continuing care funded by LAs. Moreover, the applicability of the model (in collaboration with the Department of Health) will be extended by integrating other types of care, such as hospital care and re-ablement. The current model is limited to only two types of care which can restrict local authorities that have residents moving within and between other types of care.

This work was performed using data provided by London PCTs. There are important differences between the London PCTs' populations in terms of population size, type of care, care group, age and ethnicity. This diversity would be unlikely in the rest of the country, in rural areas in particular. The results reported here cannot be generalised directly, however the models are generic and could be adapted to different settings.

Provided that the data were available, the models could also be extended in a way similar to [6] to include patient attributes such as ethnicity and evaluate their effect if any on survival patterns.

## List of abbreviations

NHS: National Health Service; LoS: Length of Stay; CC: Continuing Care; LTC: Long-Term Care; LA: Local Authority; PCT: Primary Care Trust; ILTC: Institutional Long-Term Care; HSCMG: Health and Social Care Modelling Group; CSED: Care Services Efficiency Delivery; FLoSC: Forecasting Length-of-Stay and Cost; WCC: World Class Commissioning; PF: Physically Frail; PDA: Physically Disabled Adult; OMH: Organic Mental Health; FMH: Functional Mental Health; LD: Learning Disability; ID: Identifier.

## Competing interests

The authors declare that they have no competing interests.

## Authors' contributions

SC, ED, and TJC conceived and designed the study in collaboration with the NHS London Procurement Programme (LPP). TJC obtained funding. SC, ED, TJC and PHM analysed and interpreted the data. SC, ED, and TJC applied the model. SC, ED, TJC, PHM and ST validated the models and discussed the results. SC drafted the manuscript. ED helped draft the manuscript. TJC, PHM and ST revised the manuscript critically for important intellectual content. All authors provided the final approval of the current version.

## Pre-publication history

The pre-publication history for this paper can be accessed here:

http://www.biomedcentral.com/1472-6963/11/155/prepub
